# EMPOWERING ADULTS SEEKING RECOVERY FROM SUBSTANCE AND OPIOID USE DISORDER IN THE DEEP SOUTH

**DOI:** 10.1093/geroni/igad104.2516

**Published:** 2023-12-21

**Authors:** Michael Buxton, Rebecca Allen, M Lindsey Jacobs, Brian Cox, Sarah Holditch

**Affiliations:** University of Alabama, Tuscaloosa, Alabama, United States; The University of Alabama, Tuscaloosa, Alabama, United States; University of Alabama, Tuscaloosa, Alabama, United States; Alabama Research Institute on Aging, Tuscaloosa, Alabama, United States; The University of Alabama, Tuscaloosa, Alabama, United States

## Abstract

Substance and opioid use disorder may be missed due to lack of consideration of physical, social, and emotional changes in midlife and later adulthood. This presentation uses data from a Graduate Psychology Education grant to examine predictors of alcohol use and misuse among treatment-seeking and primary care patients. Across three sites, 237 individuals completed behavioral health assessments; a rural (n = 54) and an urban federally qualified health center (n = 59), and a state-certified residential rehabilitation facility (n = 124). Participants ranged from 18 to 70 years of age (M = 40.05, SD = 1.75). Approximately 49.4% were female and 72.2% were non-Hispanic White, 23.3% African American, and 4.6% other. More than 28% of individuals had less than a high school education, and 38.4% reported a high school education or GED. Using the TAPS-II, 34.2% of participants reported alcohol use, and 13.5% reported prescription opioid misuse. Logistic regression analyses revealed that older age, prescription opioid misuse, and depression were significantly associated with alcohol use, χ2 (7) = 18.04, p = 0.12, while gender, report of adverse childhood experiences, and anxiety were not. Holding age, gender, and experiences constant, prescription opioid misuse had a significant effect on the probability of alcohol use, B = 1.092, SE = .429; Wald = 6.496, p = 0.01. The odds of alcohol use increased by a factor of 2.98 for individuals misusing prescription opioids. Treatment for problematic alcohol use must consider contextual influences including age, prescription opioids, and depression to implement effective evidence-based treatment.

